# Identification of Ferroptosis-Associated Long Noncoding RNA Prognostic Model and Tumor Immune Microenvironment in Thyroid Cancer

**DOI:** 10.1155/2022/5893998

**Published:** 2022-07-20

**Authors:** Yongjian Lin, Fu Gan, Xiaoyu He, Huachu Deng, Yong Li

**Affiliations:** ^1^Department of Gastrointestinal and Gland Surgery, The First Affiliated Hospital of Guangxi Medical University, Nanning 530021, China; ^2^Department of Urology Surgery, The Affiliated Hospital of Youjiang Medical University for Nationalities, Baise 533000, China; ^3^Department of Thoracic Surgery, The Shenzhen Baoan District Songgang People's Hospital, Shenzhen 518105, China; ^4^Department of Pharmacy, Wuhan Fourth Hospital, Wuhan 430033, China

## Abstract

**Background:**

Thyroid cancer (TC) is a rapidly increasing incidence of endocrine malignancies, occupying 3% of new cancer incidence, of which 10% has a heterogeneous prognosis. Ferroptosis is a form of cell death distinct from apoptosis, which involves antitumor drug-related research. Long noncoding RNAs (lncRNAs) could affect cancer prognosis by regulating the ferroptosis; thus, ferroptosis-associated lncRNAs are emerging as prospective biomarkers for cancer therapy and prognosis. However, the prognostic factors of ferroptosis-associated lncRNAs in this solid tumor and their mechanisms remain unknown.

**Methods:**

The TC lncRNA data were extracted from RNA sequencing files of The Cancer Genome Atlas (TCGA). Then, we performed a two-cluster analysis and grouped 502 patients with TC in a 7 : 3 ratio. Both the least absolute shrinkage and selection operator (LASSO) regression and Cox regression analysis were conducted to create and validate the ferroptosis-associated lncRNA prognostic model (Ferr-LPM). Based on the median Ferr-LPM-based risk score (LPM_score) of the training cohort, we categorized patients into high and low LPM_score groups, which were then subjected to prognostic correlation and difference analysis. We also created a nomogram and assessed its predictive ability. Furthermore, immune-related mechanisms were investigated by analyzing the tumor immune microenvironment (TIME) and applying algorithms such as CIBERSROT.

**Results:**

We built a highly accurate nomogram to promote the clinical applicability of Ferr-LPM. The area under the receiver operating characteristic curve (AUC-ROC) reached above 0.9. Survival analysis suggested that when the Ferr-LPM score was higher, the overall survival (OS) of patients within this group was shorter. Meanwhile, we found a strong association between Ferr-LPM and TIME. Interestingly, the LPM_score was inversely proportional to the tumor purity but positively related to immune checkpoint blockade (ICB) response.

**Conclusion:**

We constructed a novel ferroptosis-associated lncRNA nomogram that could highly predict the prognosis of TC patients. Ferroptosis-associated lncRNAs might possess potential functions in regulating TIME, and lncRNAs provide TC patients with new prognostic biomarkers and therapeutic targets.

## 1. Introduction

Thyroid carcinoma is a prevalent endocrine malignancy for adolescents and young adults (AYAs) (aged 15-39 years), with a continued rise in incidence worldwide over the past decade, accounting for 3% of new incidences of all carcinomas each year [1]. It includes papillary, follicular, medullary, and undifferentiated carcinoma, amidst which papillary thyroid cancer (PTC) accounts for approximately 80-85% of all differentiated thyroid cancer [2]. The vast majority of cases achieved a better prognosis after surgical resection, iodine-131 (I), and thyroid hormone replacement therapy. At the same time, 6-20% of patients still have the characteristic of recurrence, drug resistance, and aggressiveness. They ultimately progress into radioactive iodine refractory (RAIR) status, only less than 50 percent of patients achieve an overall survival of 3 years [3]. Therefore, it is of paramount significance to find reliable biomarkers that could identify individuals with worse prognoses and optimize clinical prediction models from the perspective of ferroptosis patterns.

Ferroptosis is an iron-dependent form of nonapoptotic cell death. In general, the ferroptosis-mediated cell death depends on levels of peroxidized polyunsaturated fatty acids (PUFAs) and activation of intracellular reactive oxygen species (ROS) [4]. In the last decade, investigations of anticancer drugs targeting ferroptosis have developed rapidly. Iron uptake has been demonstrated to be implicated in cellular metabolism, tumor microenvironment (TME), and immunosuppression. For example, the process of ferroptosis in cancer cells could expose antigens and thus enhance the immunogenic TME and immunotherapeutic effects [5]. It follows that the ferroptosis induction can suppress cancer cells and delay cancer progression, thus controlling prognosis in patients. Indeed, it is necessary to identify more ferroptosis-associated prognostic markers to guide thyroid cancer treatment and reduce the individual burden of heterogeneous biological behavior. However, various immune cells and complex molecular constituents also affect the mechanisms of ferroptosis on thyroid cancer cells, and the clinical significance of TC needs further investigation.

Long noncoding RNAs (lncRNAs), as transcripts in the genome sequence, involve at least 200 nucleotides [6]. It is found as the “dark matter” of the genome that regulates RNA with low expression, instability, and specificity. Although lncRNAs have only low or no protein-coding potential, they are still engaged in various biological processes, including specific regulation of transcription, translation, and posttranscriptional mRNA control. And the abnormal expression of lncRNAs acts as tumor suppressors in various human cancers. For example, lncRNA HOTAIRM1, a novel tumor-dependent prognostic marker in PTC, is significantly downregulated in PTC tissues [7]. Thus, lncRNAs could affect the epigenetic properties of many tumors. Mao et al. revealed that the functions of TC ferroptosis function are partially mediated by lncRNAs through cell line sequencing and animal experiments [8]. Furthermore, many lncRNAs are detected and analyzed in body fluids, which makes it possible for lncRNAs to serve as promising biomarkers in circulating blood of TC. Chen et al. investigated the effect of lncRNA-HOTAIR overexpression on thyroid tissues by reverse transcription-polymerase chain reaction (RT-PCR) [9]. At present, most studies only assessed individual lncRNAs, whereas tumor ferroptosis is based on widely distributed numerous genes and highly coordinated modification. Hence, it is an essential insight that ferroptosis-associated lncRNAs affect the heterogeneous prognosis, TME remodeling, and immunotherapeutic response in TC.

The antitumor effects of ferroptosis metabolism are intimately associated with the TME, which includes tumor tissue vascularization, fibroblasts, cancer cells, immune cells, extracellular matrix, and various signaling molecules. Apart from cell biology factors, acidification and hypoxia mediate cellular selection for ferroptosis resistance and apoptotic potential in solid tumors. In the hypoxic TME, tumor evasion could promote ferroptosis resistance, reduce phagocytosis, and affect immunogenic ferroptosis. Liu et al. found that Fe^2+^ from hypoxic cancer cells, by metabolic coupling with the labile iron pool (LIP), coregulates mitochondrial iron homeostasis in cancer cells to promote invasion [10]. Besides, TME also intervenes in cancer patients' survival through tumor-infiltrating immune cells (TIICs). Bergdorf et al. demonstrated that dendritic cells and neutrophils could promote lymph node (LN) metastasis and affect prognosis through the LAG3-mediated immunosuppression in PTCs [11]. The latest ICB synergetic triple stimuli-activated nanoimmunotherapy has represented that more potential immune regimens effectively improved the overall response of cancer patients [12]. In summary, ferroptosis-associated lncRNAs have significant potential to become prognostic assessment markers and provide immunotherapeutic strategies for TC.

Recent research has shown that lncRNAs were key regulators of tumor progression at the epigenetic levels, such as the modification, silencing, recruitment, and scaffold functions [13]. Many tumor biomarkers and checkpoints rely on interaction with other cellular macromolecules (e.g., DNA, RNA, and protein) and either creating a substrate or inhibitory protein effectors for protein function. This study focused on ferroptosis-associated lncRNAs to confirm the Ferr-LPM and TIME signature in TC. With TCGA database, we performed bioinformatics methods to mine the TC genome. After coexpression screening of ferroptosis-associated lncRNAs, the LASSO-penalized Cox regression, and univariate and multivariate Cox regression analyses, we first constructed a prognostic model of ferroptosis-associated lncRNAs and a novel and efficient nomogram. Finally, the related intratumoral immune landscape, immune genes, and immunotherapy were discussed.

## 2. Materials and Methods

### 2.1. Acquisition of the Thyroid Dataset

To obtain TCGA thyroid cancer cohort (*n* = 568), we downloaded uniformly processed RNA sequencing and corresponding clinical follow-up data from the Genomic Data Commons Data Portal (https://portal.gdc.cancer.gov/). We excluded three patients with incomplete survival data and five duplicates. Eventually, 502 patients were left to be enrolled, which were randomly assigned to the training and test cohorts via the caret package in R. Ferroptosis-disease associations were collected from the first manually collated database of ferroptosis (FerrDb).

### 2.2. Enrichment Analysis of Ferr-Associated DEGs

Utilizing the R package “limma” to identify ferroptosis-associated differentially expressed genes (DEGs) with |log2 (fold change) | ≥1. The “clusterprofiler” package in R was adopted for the Kyoto Encyclopedia of Genes and Genomes (KEGG) to enrich potential functional pathways according to Gene Ontology (GO). Furthermore, the potential functional pathways between TCGA transcriptome DEGs were enriched by the Gene Set Enrichment Analysis (GSEA), which performed a thousand gene permutations. The human genome annotation files have been uploaded to the GSEA website (https://www.gsea-msigdb.org/gsea).

### 2.3. Development of Ferr-Associated lncRNAs

We downloaded 259 transcriptomic RNAs associated with ferroptosis from FerrDb and screened ferroptosis-associated lncRNAs in the TC samples, with correlation coefficient filtering criteria set at >0.7 and *P* < 0.001. The R package “reshape2” could visualize the coexpression networks between lncRNAs and ferroptosis-related genes. Venn diagram took the common portions to identify candidate genes for the Ferr-LPM. To further screen for ferroptosis-associated lncNRAs on prognosis, we performed univariate Cox regression analysis for overall survival. Based on the differential expression of ferroptosis-associated lncRNAs, we performed unsupervised cluster analysis on TC samples and conducted survival analysis on the clustering results. In addition, we analyzed the correlation between clinical data and clustering results and calculated the semi-inhibitory concentration (IC50) values for prognostic outcomes under common chemotherapy regimens.

### 2.4. Ferr-LPM and Nomogram in TC

We constructed a least absolute shrinkage and selection operator (LASSO) regression model utilizing prognosis-related DEGs. Ferroptosis-associated lncRNA prognostic model was stratified based on risk scores = ∑(exp(lncRNAs)∗*β*), where exp denoted the expression of lncRNAs in tumor samples and *β* represents its coefficient. Patients with TC above and below the median risk score of the training group were included in the high and low LPM_score group. Afterward, the receiver operating characteristic (ROC) curves were applied to estimate the predictive efficacy of the Ferr-LPM, and then, scatter plots of risk scores were drawn for each cohort. To facilitate the construction of forest plots, we selected the “glmnet” and “survival” R packages to carry out Cox regression analyses of possible independent or multiple prognostic factors. The correlation heatmap of clinical features was derived from the stratification analysis of the whole TC samples. We integrated the prognostic factors of TC to present a nomogram and validated it by employing the concordance index and calibration curves. Use of this nomogram predicted 1-, 3-, 5-, and 10-year OS in TC patients.

### 2.5. Immunological Analysis and Related Gene Expression

Based on the LM22 immune signature and 1000 permutations, we systematically analyzed human immune infiltrating immune cell profiles of 502 TC patients by the CIBERSORT algorithm. The correlation module drew the scatterplots between Ferr-LPM and TIIC. ESTIMATE was used to calculate immune and stromal fractions in carcinoma tissue. The stromal-immune-ESTIMATE scores indicated the proportion of immune/tumor components in each patient. For the TME, higher scores indicate relatively high immune/stromal cell content and low tumor purity. Meanwhile, the TIMER, CIBERSORT, QUANTISEQ, MCPcounter, XCELL, and EPIC algorithms were compared for both cohorts to assess immune compositional characteristics and predict immune infiltration. Besides, we used ssGSEA to quantify the infiltration and immunological function of immune cell subgroups by the GSVA R package. According to the two subgroups of Ferr-LPM, we used the box plot to reveal the differential expression of 47 immune checkpoints, including programmed death 1 (PD-L1), cytotoxic T-lymphocyte associated protein 4 (CTLA4), B and T lymphocyte attenuator (BTLA), T cell immunoreceptor with Ig and ITIM domains (TIGIT), and lymphocyte activating 3 (LAG3). In this study, the Immune Cell Abundance Identifier (ImmuCellAI) helped TC samples to predict the response of ICB therapy with the examination.

### 2.6. Statistical Analysis

All data were statistically analyzed with the R software (version 4.0.3), and nominal *P* value < 0.05 with two-tailed had statistical significance. False discovery rate (FDR) was used to multiply correct *P* values in DEGs, GO, and KEGG analyses. Pearson correlation analysis is the most appropriate for continuous, normal, and linear data, while Spearman correlation analysis is not as efficient as the former. Gene expression levels, TIIC distribution, and tumor purity were analyzed using the two-sample Wilcox test. The Chi-square test analyzed stratified differences in clinical traits among the Ferr-LPM subgroups. Kaplan-Meier analysis and chi-square test were adopted to compare the overall survival between the LPM_score groups. Identification of independent prognostic factors was tested using Cox regression analysis for both univariate and multivariate factors.

## 3. Result

### 3.1. Functional Enrichment Analysis of Ferroptosis-Associated DEGs

To explore potential ferroptosis regulatory signaling pathways, we identified 18 activations and 9 inhibitions of ferroptosis-associated DEGs. Then, we performed GO analysis to enrich the DEG-related functional pathways (Additional file 2: Figure S2(a)). Biological process (BP) involved in response to oxidative stress, regulation of MAP kinase activity, and apoptotic signaling pathway. Molecular function (MF) principally regulated iron ion binding, growth factors, heme, binding, and the generation of oxidoreductase and NAD(P) H oxidase. Cellular components (CC) mainly constitute the apical plasma membrane, focal adhesion, cell-substrate junction, and apical part of the cell. KEGG enrichment analysis demonstrated significant upregulation of differential genes mainly in central carbon metabolism, Th17 cell differentiation, serotonergic synapse formation, MAPK signaling pathway, cytokine-receptor interaction, cellular senescence, and microRNA synthesis in cancer (Additional file 2: Figure S2(b)).

### 3.2. Ferroptosis-Associated lncRNAs

**Figure 1:**
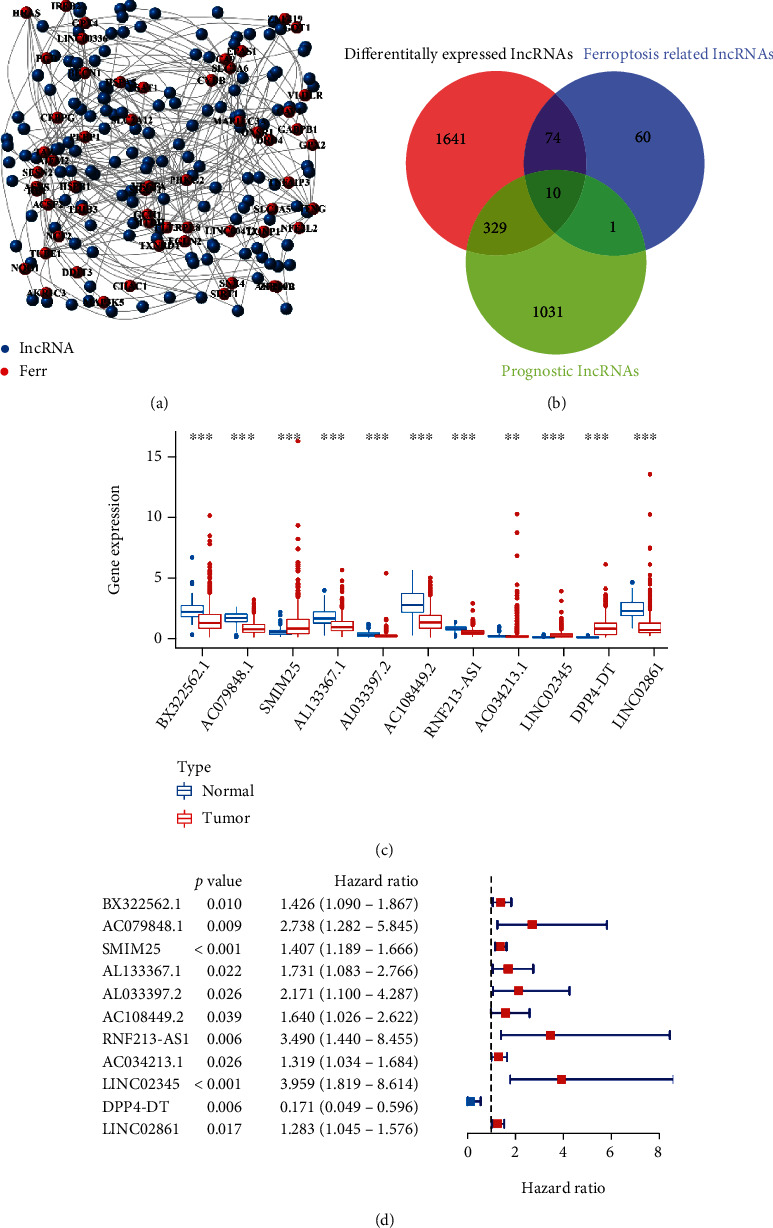
Identification of Ferr-associated prognostic lncRNAs in TC. (a) Coexpression network diagram of the 60 Ferr-associated genes and 145 lncRNAs. (b) Venn diagrams to identify common lncRNAs of differentially expressed lncRNAs, Ferr-associated lncRNAs, and prognostic lncRNAs. (c) The 11 overlapping lncRNAs were differentially expressed in normal and tumor tissues. (d) Forest plot of univariable regression analysis results of the 11 selected Ferr-associated lncRNAs. The “∗∗” represents the statistically significant *P* value < 0.01, and ^∗∗∗^*P* < 0.001.

According to TCGA dataset with 58 normal and 510 tumor sample, we screened 145 ferroptosis-associated lncRNAs by Wilcox text (*P* < 0.001, ∣Cor | >0.7), presenting an integrated landscape of 145 associated lncRNAs and ferroptosis interactions in the coexpression network (Figure 1(a)). Eleven ferroptosis-associated prognostic lncRNAs were determined from univariate Cox regression analysis (Figures 1(b) and 1(d)), including BX322562.1, AC079848.1, SMIM25, AL133367.1, AL033397.2, AC108449.2, RNF213-AS1, AC034213.1, LINC02345, DPP4-DT, and LINC02861 (Additional file 3: Table S1). Ten target genes were located within the central intersection of the Wayne diagram. Interestingly, lncRNA AC034213.1 was only within the intersection of two sets, which were ferroptosis-associated and prognostic lncRNAs (Figure 1(c)).

### 3.3. Cluster Analysis of Ferroptosis-Associated Prognostic lncRNAs

**Figure 2:**
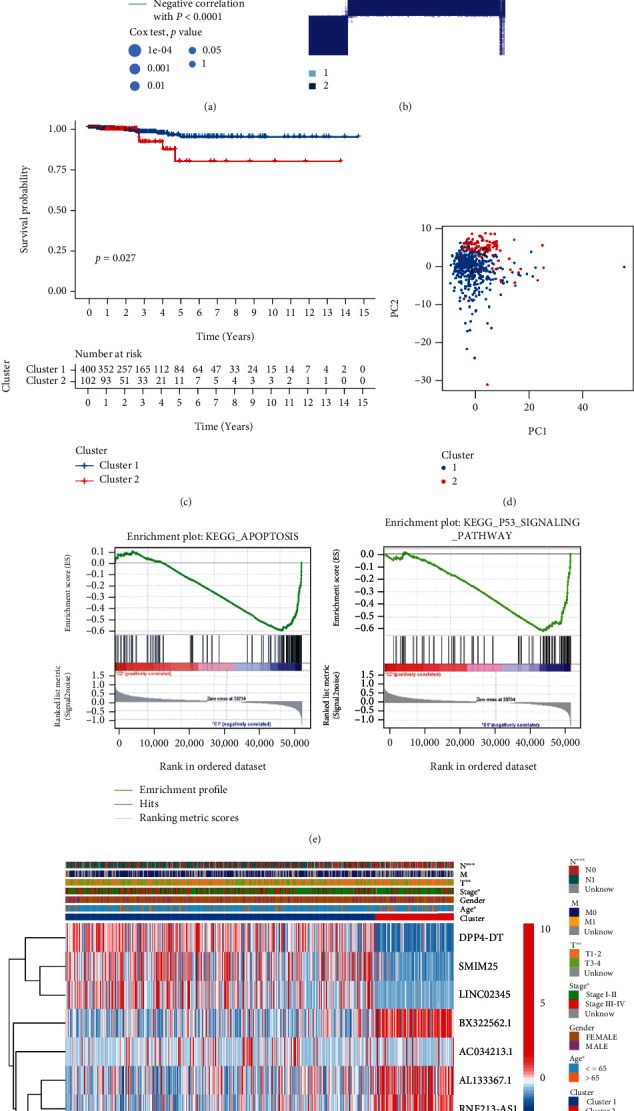
Unsupervised cluster analysis of Ferr-associated prognostic lncRNAs. (a) Interactions among lncRNAs in TC. The line connecting the genes represents their interaction, with the line thickness indicating the strength of the association between genes. Green and pink represent negative and positive correlations, respectively. (b) Consensus clustering matrix for *k* = 2. (c) K-M survival curves of overall survival (OS) in C1 and C2 clusters. (d) PCA analysis shows a discriminable dimension between the two clusters. (e) GSEA results show significant enrichment of apoptosis pathway and p53 pathway in cluster1. (f) Differences in clinicopathologic features and expression levels of lncRNAs between the two clusters.

Consistency clustering analysis distinguished two subgroups of thyroid cancer patients with similarity and heterogeneity (Figure 2(b)). Principal component analyses (PCAs) indicated discriminable dimensions between cluster1 and cluster2 groups, verifying that they had different PCAs (Figure 2(d)). We performed survival analysis, and the K-M cures revealed that the C1 group had a more prolonged OS and a better prognostic advantage than the C2 group (log-rank sum test, *P* = 0.027, Figure 2(c)). Additionally, comparing the clinicopathological features of two subtypes, we found an association between overexpression of lncRNAs and clinicopathological characteristics, which were plotted as a heatmap (Figure 2(f)).

### 3.4. Characteristics of the TME in Distinct Clusters

**Figure 3:**
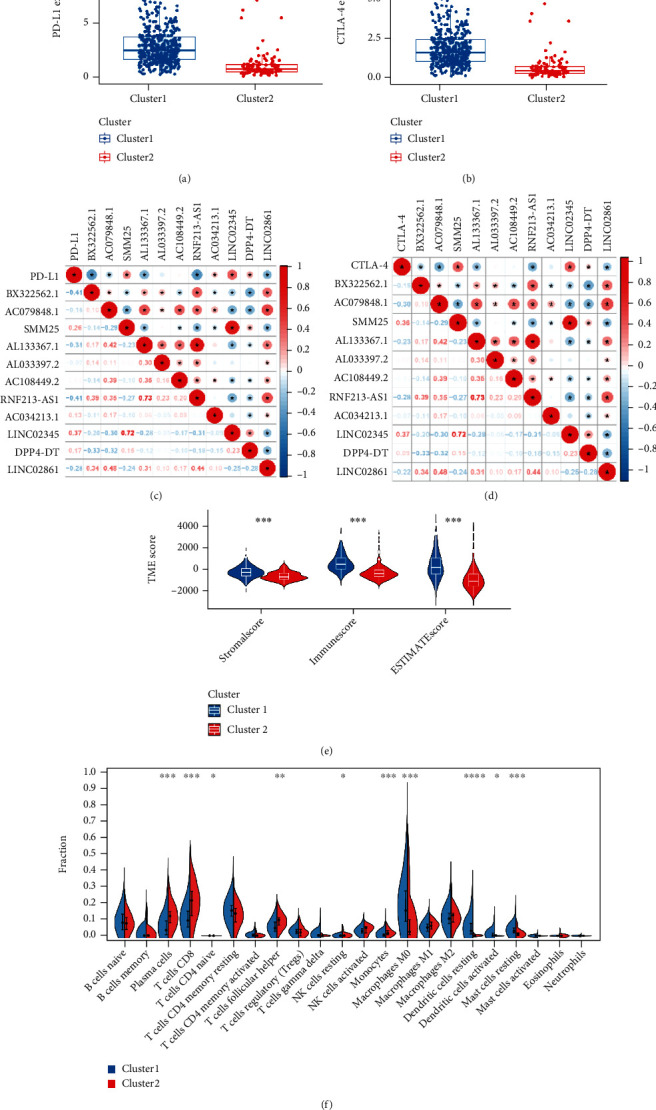
Immunity analysis of clusters. (a, b) Differential expression analysis of immune checkpoints: (a) PD-L1 and (b) CTLA4. (c, d) Correlation plots between 11 Ferr-related lncRNAs and PD-L1 or CTLA4. The “∗” represents the statistically significant *P* value < 0.05, ^∗∗^*P* < 0.01, ^∗∗∗^*P* < 0.001, and ^∗∗∗∗^*P* < 0.0001. Red color represents positive correlation, while blue color represents negative correlation. The depth of colors represents the correlation value, ranging from-1 to 1. (e) Correlation between clusters and immune or stromal scores. (f) Violin diagram of TIICs distribution for two clusters.

The clustering GSEA results revealed significant enrichment in the majority of immune and tumor-associated signaling pathways, including NOD-like receptor, JAK-STAT, P53, natural killer cell-mediated cytotoxicity, MAPK, Fc-*γ*R-mediated phagocytosis, and RIG-I-like and TOLL-like receptor signaling pathways (Figure 2(e), Additional file 2: Figure S2(c)-(j)). Given these results, we evaluated the relationship between two clusters and 22 subpopulations of human immune cells. A considerable difference was found in the infiltration landscapes between the two clusters (Figure 3(f)). Simultaneous coexpression analysis between two critical molecular markers of tumor immunotherapy and ferroptosis-associated prognostic lncRNAs showed that nine lncRNAs were significantly associated with PD-L1 and eight lncRNAs were closely related to CTLA4 (Figures 3(a)–3(c)). Additionally, the scores correlated with the immune/stromal components ratio indicated patients in cluster1 had high immune content and low tumor purity, and the opposite was true for cluster2 (*P* < 0.05) (Figure 3(e)). These results demonstrated a significant association between our target lncRNAs and tumor immunity.

### 3.5. Constructing and Validating a Prognostic Model for TC

The prognosis of patients with TC could be evaluated by survival times and survival status. We performed the LASSO algorithm and Chi-square test on the 11 Ferr-associated lncRNAs obtained from previous univariate Cox analysis. According to the minimum partial likelihood of deviance (*λ* = 6), the ferroptosis-associated lncRNA prognostic model was constructed as follows (Additional file 4: Table S2): risk score = (0.7630∗expression of AC079848.1) + (0.1293∗expression of SMIM25) + (0.3308∗expression of AL033397.2) + (0.0006∗expression of AC108449.2) + (0.1072∗expression of AC034213.1) + (0.0766∗expression of LINC02861).

**Figure 4:**
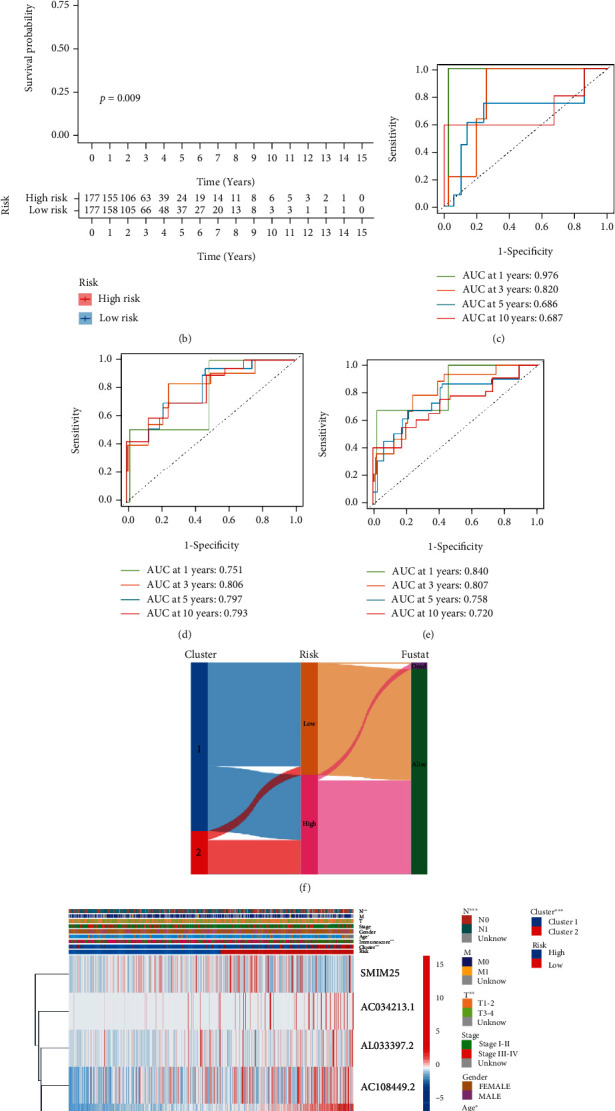
Construction and validation of Ferr-LPM. (a, b) The K-M curves showed that the high LPM_score group had inferior OS in the test set (a) and training set (b). (c–e) ROC curves of 1, 3, 5, and 10-year OS for the test set (c), training set (d), and total set (e). (f) Alluvial diagram of gene clusters in groups with different LPM_score subtypes and survival status in TCGA cohort. (g) Stratified analysis of Ferr-associated lncRNAs, risk scores, and clinical factors. (h) Independent prognostic analysis of Ferr-LPM.

To further elucidate the role of Ferr-LPM, we randomized TC patients into training and test sets and then plotted the survival status and time distribution of patients with different risk scores (Additional file 5: Table S3, Additional file 6: Figure S3(a)-(d)). Heatmaps showed the expression of ferroptosis-associated lncRNAs (Additional file 6: Figure S3(e), (f)). With the growth of the LPM_score, the overall survival of patients decreased and mortality increased. Kaplan-Meier survival curves were fitted to the training and test sets, respectively (Figures 4(a) and 4(b)). The figures exhibited that the overall survival of TC patients with low LPM_score was significantly prolonged in both the training and test sets. In general, the performance of a predictive model was represented by the AUC value, with a larger AUC-ROC indicating higher accuracy (Figures 4(c)–4(e)). Overall, the Ferr-LPS has superior predictive power to standard clinicopathological features.

### 3.6. Prognostic Analysis and Nomogram Construction

**Figure 5:**
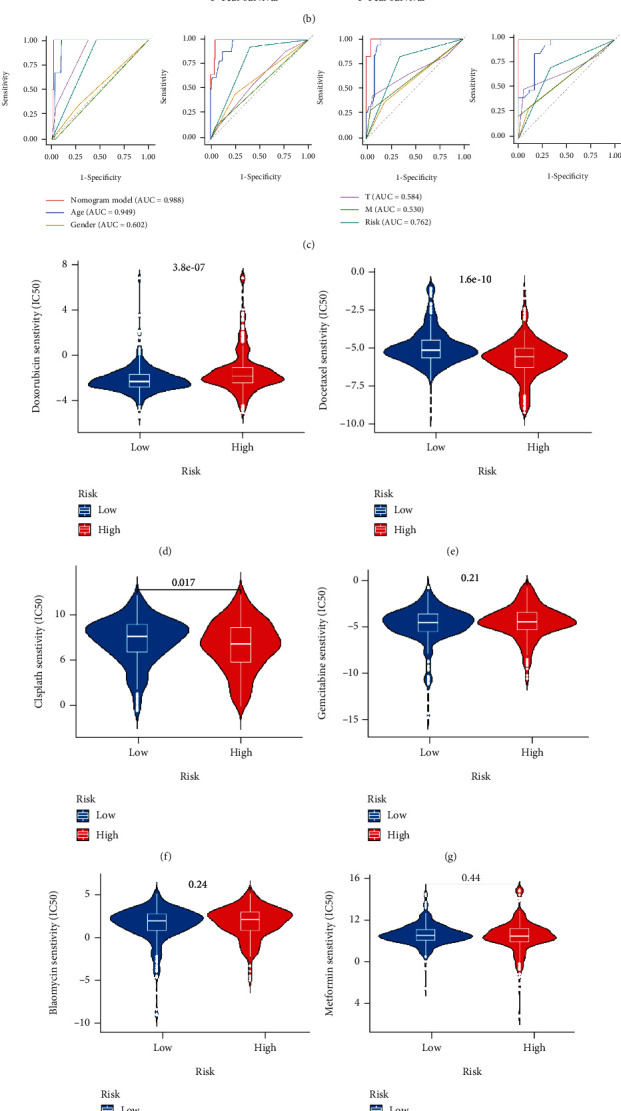
Construction of a nomogram and comprehensive validation of Ferr-LPM. (a) Nomogram for both clinicopathological factors and Ferr-LPM. (b) Calibration curve of the nomogram for predicting 1-, 3-, 5-, and 10-year OS. (c) The ROC curves for 1-, 3-, 5-, and 10-year nomograms. (d–k) The relationship between LPM_score and chemotherapy sensitivity.

Both univariate and multivariate analyses illustrated that the LPM_score could serve as an independent prognostic factor for TC (Figure 4(h)). Besides, we utilized stratification analysis to test whether the Ferr-LPM maintained predictive capability in different subgroups (Figure 4(g)). The results indicated that patients with high LPM_score exhibited pronounced OS decline in different subgroups, including older (>65 years, *P* = 0.039) and younger (≤65 years, *P* = 0.036), female (*P* = 0.013) and male (*P* = 0.03), advanced- (stages I-II, *P* = 0.018) and early-stage (stages III-IV, *P* = 0.006), M0 stage (*P* = 0.005), N0 (*P* = 0.03) and N1-3 stage (*P* = 0.002), T1-2 (*P* = 0.008), and T3-4 stage (*P* = 0.026) (Additional file 7: Figure S4). Moreover, the Ferr-LPM was adept in discriminating TC patients with differential immune-score (Additional file 8: Figure S5(a)–(c)). Combining the above results, we established a nomogram incorporating clinicopathological characteristics and Ferr-LPM (Figure 5(a)). The ROC-AUC of 1, 3, 5, and 10 years for the nomogram were 0.988, 0.985, 0.992, and 0.999, respectively (Figure 5(c)). The calibration curves further illustrated the accuracy of the survival prediction nomogram (Figure 5(b)). Prognosis-related chemotherapy sensitivity analysis had a lower IC50 for docetaxel in the high LPM_score group, while doxorubicin had a significantly lower IC50 in the low LPM_score group (Figures 5(d) and 5(e)). The IC50 values of cisplatin, gemcitabine, bleomycin, metformin, etoposide, and sorafenib were retarded (Figures 5(f)–5(l)).

### 3.7. Immunization and Therapeutic Evaluation

**Figure 6:**
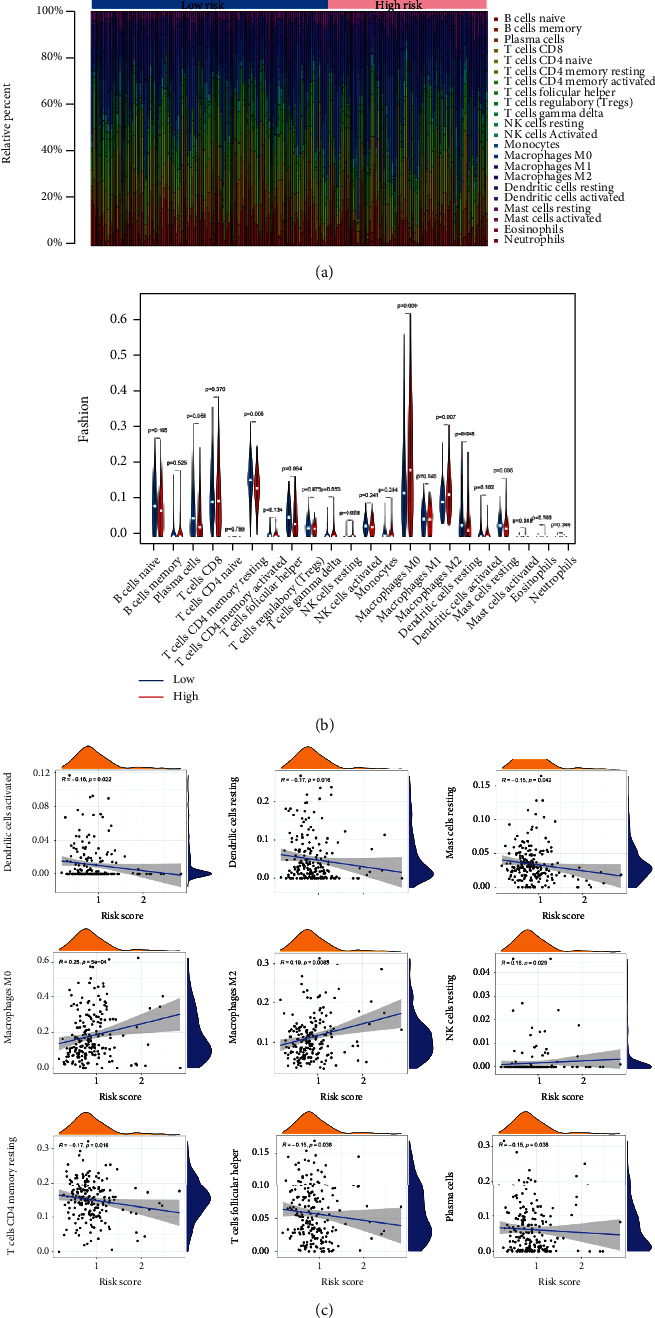
The immune cell infiltration landscape in thyroid cancer. (a) Barplot of the tumor-infiltrating cell proportions. (b) Violin diagram of the tumor-infiltrating cell proportions. (c) Correlation between LPM_score and immune cell types.

**Figure 7:**
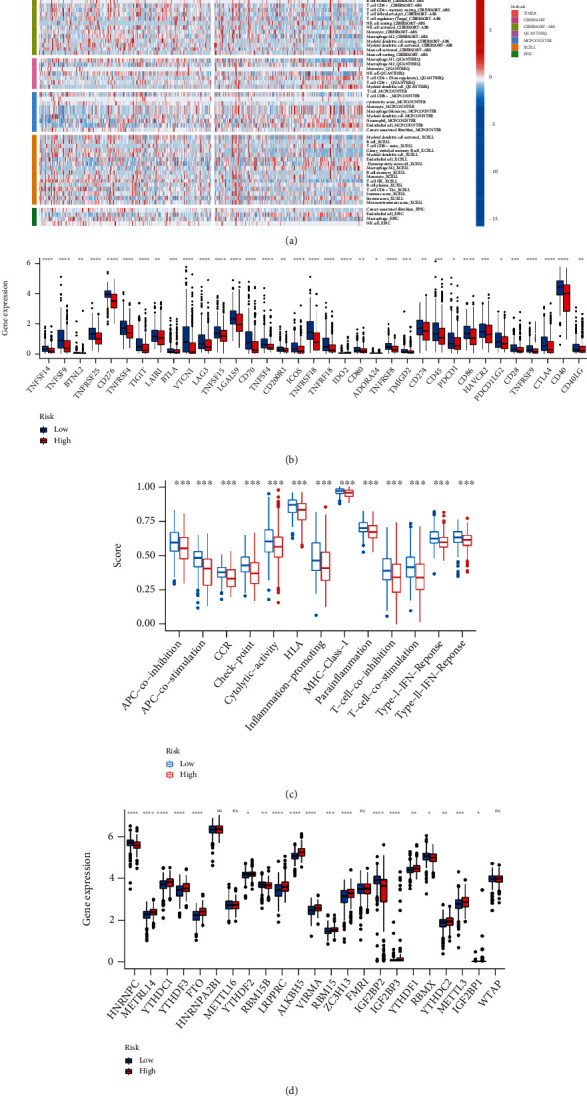
Immunity and gene expression. (a) Heatmap for immune responses based on 7 algorithms between high- and low-risk groups. (b) Expression of immune checkpoints in the high and low LPM_score groups. (c) ssGSEA for the association between immune cell subsets and related functions. (d) The expression of m6a-related genes in high and low LPM_score groups.

CIBERSORT could impute the fraction of 22 immune infiltrating cells in TC by the deconvolution algorithm. Our investigation revealed that compared with the low LPM_score group, M0 (*P* = 0.009) and M2 macrophages (*P* = 0.007) were significantly activated in the high LPM_score group, whereas opposite proportions of resting memory CD4+ T cell (*P* = 0.005), resting dendritic cells (*P* = 0.048), and mast cells (*P* = 0.035) were observed (Figures 6(a) and 6(b)). TIIC-related survival curves implied the association between immune landscape with prognosis (Additional file 9: Figure S6). As shown in the scatter plot, the LPM_score was negatively linked to adaptive immune response cells, including activated dendritic cells (*R* = −0.16, *P* = 0.022), resting dendritic cells (*R* = −0.17, *P* = 0.016), resting mast cells (*R* = −0.15, *P* = 0.042), plasma cells (*R* = −0.15, *P* = 0.038), resting memory CD4+ T cells (*R* = −0.17, *P* = 0.016), and follicular helper T cells (*R* = −0.15, *P* = 0.038), while innate immune response cells were reversed, including macrophages M0 (*R* = 0.25, *P* < 0.001), macrophages M2 (*R* = 0.19, *P* = 0.0083), and natural killer cells (*R* = 0.16, *P* = 0.029) (Figure 6(c)). Based on seven algorithms, the heatmap of immune responses compared TIIC differences in LPM (Figure 7(a)). In terms of ssGSEA, quantification of enrichment scores implied that the low LPM_score group might benefit from immune cell subsets and related functions (Figure 7(c)). We observed a significant correlation between Ferr-LPM and 35 immune checkpoints (Figure 7(b)), in which the LPM_score of TC patients was inversely trended with the expression of PD-L1 or CTLA4 (Additional file 8: Figure S5(d), (e)), and the outcome reflected that patients with low scores had more immune locus and protein expression. Further, the LPM_scores also correlated markedly with the expression of N6-methyladenosine- (m6a-) related lncRNAs (Figure 7(d)). Prediction data from ImmuCellAI showed that the ICB-response group scored better on Ferr-LPM than the nonresponse group (*P* = 0.0092) (Additional file 8: Figure S5(f)).

## 4. Discussion

Accumulative studies have shown that cancer cell ferroptosis is indispensable in tumor prognosis and immune microenvironment [14]. We investigated the comediated effects of multiple ferroptosis-associated lncRNAs among thyroid cancer prognostic model and immune infiltration characteristics in this study. The immune-related analysis of clustering results suggested that Ferr-associated lncRNAs might negatively regulate immune checkpoints (PD-L1 and CTLA4). Many ferroptosis-associated lncRNAs have been explored in TC prognosis and treatment. In Ferr-LPM, lower and higher risk scores corresponded to ferroptosis patterns characterized by immune activation and depression, respectively, while revealing notably different clinicopathological features, TME quantitative immunogenicity scores, immune cell functions, and immunotherapy responsiveness. Therefore, it was the first time we constructed the ferroptosis-associated lncRNA prognostic model for thyroid cancer.

In view of the potential value in lncRNAs and ferroptosis-associated immunity, previous investigations have also built other ferroptosis-associated lncRNA models to evaluate tumor prognosis. Based on 17 ferroptosis-associated lncRNAs, Pan et al. developed a prognostic model for gastric cancer, which had AUCs of 0.747 and 0.751 in the test and control groups [15]. The AUC for lung squamous cell carcinoma- (LUSC-) related model was 0.658 at 1 year, 0.693 at 2 years, and 0.687 at 3 years. Other prognostic models have been constructed in recent years; however, the accuracy was generally under 0.8. In contrast, Ferr-LPM could improve the prognostic power of TC. Our model further helps clinicians predict outcomes and prognosis, will assist in understanding the molecular mechanisms of TC, and provide new ways for immune-targeted therapies.

Our results found that eleven lncRNAs play a key role in affecting the prognosis of TC patients. A current research discovered that SMIM25 (LINC01272) downregulation in gastric cancer contributed to the migration and invasion of cells via epithelial-mesenchymal transition (EMT) [16]. In addition. LINC01272 in lung cancer inhibits multiplication and induces programmed cell death via the miR-7-5p/CRLS1 pathway, and hypoexpression of LINC01272 corresponds to poor prognosis [17]. Meanwhile, a bioinformatics analysis found that prognosis-related immune lncRNA signature AC108449.2 could serve as potential immunotherapy targets for KIRC patients [18]. However, there was little evidence for a direct relation between DPP4-DT (DPP4 divergent transcript) and TC or between AC034213.1 and TC. Lopez-Campistrous et al. have shown that DPP4 gene silencing not only prevented PTC cell proliferation and EMT by inhibiting the MAPK pathway but also shed light on a related pathway about cell apoptosis in thyroid cancer [19]. Currently, studies on ferroptosis-associated lncRNAs are few, and we have not yet found research regarding the significance of AC079848.1, AL033397.2, and LINC02861 in TC or other tumors, particularly from the perspective of TC prognosis. Through integrating the Ferr-LPM and clinicopathological features, we have created a quantitative nomogram to intuitively evaluate the prognosis. These findings might give invaluable perspective for the future of cancer control.

Ferroptosis, being with the tumor-suppressive properties, could remove defective cells and overcome the chemoresistance of malignant tumors [20]. In cancer progression, specific gene regulation and drug induction were two aspects of the inducing ferroptosis mechanism in cancer cells. On the one hand, lncRNA expression affects TC progression and resistance in single chemotherapy regimens. After the knockdown of CCAT2, Fu et al. found a subsequent decrease in IC50 values of doxorubicin and cisplatin, thus affecting the prognosis of mesenchymal thyroid cancer. PTCSC3 is the tumor suppressor lncRNA of TC and negatively regulates STAT3/INO80 to attenuate the resistance to doxorubicin that favors prognosis. On the other hand, the various drugs (e.g., sorafenib, lorazepam, artesunate, and erastin) that induce ferroptosis are available for antitumor therapies [21], while cancer cells tend to promote survival and metastasis by resisting ferroptosis. Besides, we also found the correlation between ferroptosis-associated lncRNAs and TME. Multiple signaling pathways in the microenvironment could confer ferroptosis resistance in cancer cells, such as common hypoxia. In the TME of thyroid carcinomas, lncRNAs might facilitate tumor progression, cell migration, invasion, and angiogenesis. These evidence suggested that the downregulation of drug resistance genes and elevated chemosensitive lncRNAs presented a way for precision medicine to address heterogeneous prognosis. Ferroptosis-associated lncRNAs might offer a different molecular theoretical basis for the clinical development of novel prognostic models.

The prognostic benefits in TC patients are closely linked to TIICs in the microenvironment. The results of tumor-infiltrating immune cell analysis implied that patients with a high LPM_score would accumulate more M2 macrophages with immune-suppressive phenotype, leading to a worse prognosis. The phenomenon was also found in the histological investigation of 90 PDTC cases, and an increased number of TAMs was correlated with cystic invasion, extrathyroidal growth, and reduced survival. Resting memory CD4 T cells, resting DC cells, and resting mast cells were reduced in this group. One of these mechanisms is the downregulation of B7H1 expression, which is acquired by cancer cells during tumorigenesis, and the metastases became apparent when B7H1 ligand-rich T cells were depleted. As a result, the immune response was rarely sufficient to eliminate tumors, we hypothesized that tumor-associated macrophages (TAMs), CCR, checkpoint, and IFN responses might contribute to the “immune escape.” DCs are involved in innate and adaptive immunity. They have “immune surveillance” effects such as antigen-presenting cell (APC) and proinflammatory. Early studies have shown that TC patients with dense infiltration of DCs have a favorable prognosis regardless of morphological and clinical features. Our low LPM_score group tended to be immunologically “immune surveillance” and was more likely to promote a superior prognosis. In a similar LUSC risk scoring, the ferroptosis prognostic model was interlinked with “immune escape” features, suggesting major therapeutic directions to restore or enhance “immune surveillance,” such as ferroptosis inducers with immune checkpoint inhibitors [22]. With the support of comprehensive immunoassay, ferroptosis-associated lncRNAs in gastric, hepatocellular, and bladder cancers have achieved remarkable results in prognostic models and TIME. So far, a combined strategy of the stromal score and clinicopathological patterns is available to predict progression-free survival (PFS). The next study should validate the viability of immunological characteristics of lncRNAs as biomarkers for TC.

Immunotherapy remains a nonconventional strategy for TC, with guidelines considering novel agents for patients with inoperable, RAI, or progressive distant metastases. Indeed, immunotherapy has emerged as a promising weapon for advanced TC, and combination ICB therapies targeting CTLA4 or PD1-PDL1 have been tested and achieved high response rates and real long-term efficacy, even benefiting the immunotherapy-resistant patients. Interestingly, some patients with low immune checkpoints may benefit from ICB therapy, so we believed that their tumor ferroptosis could cause immunosuppression and immunotherapy resistance. Meanwhile, the sensitivity of ICB therapy response was closely relevant to ferroptosis-associated lncRNAs and strongly coupled to prognosis. Due to the well-predicted results of the Ferr-LPS, the combination of ferroptosis-associated lncRNAs and immune checkpoint might provide considerable value for predicting the efficacy of ICB therapy. We further validated the accuracy of the Ferr-LPM prediction results with m6a-related lncRNA, such as the FTO protein correlated with lymph node metastasis and tumor grading and METTLE3, which regulated neutrophils and was intimately associated with malignant progression in PTC [23]. These findings suggested that ferroptosis-associated lncRNAs combined with immune checkpoints contribute to improving the prognosis rate of TC and provide references for clinical applications, such as biomarkers for prognosis, stratification markers, drug sensitizers, and therapeutic targets.

However, several limitations of our study remained. First, this is a preliminary study based on bioinformatics tools, combining real-world samples and complimentary validation sets will allow design rigor. Next, restricted by public databases, we need tumor/normal samples from other databases to reduce serendipity. Then, the ferroptosis process is not a specific mechanism of TC, and ferroptosis-associated lncRNAs mediating TIME are still stuck in our correlation and algorithms. Finally, algorithms to calculate immune cell abundance are not as accurate as immunofluorescence, and biochemical experiments should be required.

## 5. Conclusion

In summary, we initially explored the status of ferroptosis in TC and its relevance to TC prognosis. A Ferr-LPM was created based on six lncRNAs related to TC and validated for specificity and sensitivity. This risk scoring model exhibited promising progress in predicting immune infiltration and checkpoint response, which will provide novel strategies for ICB therapy of TC.

## Data Availability

The data used to support the findings of this study are included within the article.

## Supplementary Materials

Supplementary 1 Additional file 1: Figure S1: the flowchart of our study.

Supplementary 2 Additional file 2: Figure S2: functional enrichment analysis. (a, b) GO and KEGG analyses for ferroptosis-associated differentially expressed genes. (a) GO and (b) KEGG. (c–j) Several abundant immunologic characteristics of cluster1 and cluster2.

Supplementary 3 Additional file 3: Table S1: the eleven ferroptosis-associated prognostic lncRNAs.

Supplementary 4 Additional file 4: Table S2: the coefficients of the LASSO regression model in this study.

Supplementary 5 Additional file 5: Table S3: the baseline of training and testing sets.

Supplementary 6 Additional file 6: Figure S3: identifications of prognostic gene signatures for the training set and test set, respectively. (a, b) Risk score distribution of TC patients in the training and test groups. (c, d) The scatter plot about the correlation between survival time and risk score. (e, f) The heatmap of six Ferr-associated lncRNAs in high- and low-risk TC patients.

Supplementary 7 Additional file 7: Figure S4: the survival curves of stratification analysis. (a, b) Age. (a) ≤65 and (b) >65. (c, d) Gender. (c) Female and (d) male. (e–i) Tumor stage. (e) Stages I-II and (f) stages III-IV, (g) M0 and (h) M1, (i) N0 and (j) N1-3, (k) T1-2, and (l) T3-4.

Supplementary 8 Additional file 8: Figure S5: tumor purity and ICB therapy response analysis. (a–c) Scattered box diagrams of immune scores, stromal scores, and ESTIMATE scores in two groups. (d, e) The differential expression analysis of two key immune checkpoints (PD-1 and CTLA4) in low- and high-risk groups. (f) The relationship between risk score and immune checkpoint blockade (ICB) therapy response.

Supplementary 9 Additional file 9: Figure S6: the survival curves of TIICs for high- and low-risk TC patients. (a) Plasma cells, (b) T cell CD8, (c) T cell gamma delta, (d) NK cells activated, (e) monocytes, (f) macrophage M0, (g) macrophage M1, (h) macrophage M2, (i) dendritic cells resting, (j) dendritic cells activated, and (k) mast cells resting.

## References

[1] Miller K. D., Fidler-Benaoudia M., Keegan T. H., Hipp H. S., Jemal A., Siegel R. L. (2020). Cancer statistics for adolescents and young adults. *Ca-a Cancer Journal for Clinicians*.

[2] Prete A., De Souza P. B., Censi S., Muzza M., Nucci N., Sponziello M. (2020). Update on fundamental mechanisms of thyroid cancer. *Frontiers in Endocrinology*.

[3] Shobab L., Gomes-Lima C., Zeymo A. (2019). Clinical, pathological, and molecular profiling of radioactive iodine refractory differentiated thyroid cancer. *Thyroid*.

[4] Jiang X., Stockwell B. R., Conrad M. (2021). Ferroptosis: mechanisms, biology and role in disease. *Nature Reviews Molecular Cell Biology*.

[5] Zhang F., Li F., Lu G.-H. (2019). Engineering magnetosomes for ferroptosis/immunomodulation synergism in cancer. *ACS Nano*.

[6] Ramilowski J. A., Yip C. W., Agrawal S. (2020). Functional annotation of human long noncoding RNAs via molecular phenotyping. *Genome Research*.

[7] Li D., Chai L., Yu X. (2020). The HOTAIRM1/mi R-107/TDG axis regulates papillary thyroid cancer cell proliferation and invasion. *Cell Death & Disease*.

[8] Mao C., Wang X., Liu Y. (2018). A G3BP1-interacting lncRNA promotes ferroptosis and apoptosis in cancer via nuclear sequestration of p53. *Cancer Research*.

[9] Chen X., Jin J., Zheng L., Sheng Y., Sun J. (2021). Correlations of HOTAIR expression with pathological stage, CT characteristics and prognosis of patients with papillary thyroid carcinoma. *Journal of BUON*.

[10] Liu S., Cao X., Wang D., Zhu H. (2022). Iron metabolism: state of the art in hypoxic cancer cell biology. *Archives of Biochemistry and Biophysics*.

[11] Bergdorf K., Ferguson D. C., Mehrad M., Ely K., Stricker T., Weiss V. L. (2019). Papillary thyroid carcinoma behavior: clues in the tumor microenvironment. *Endocrine-Related Cancer*.

[12] Bai S., Lu Z., Jiang Y. (2022). Nanotransferrin-based programmable catalysis mediates three-pronged induction of oxidative stress to enhance cancer immunotherapy. *ACS Nano*.

[13] Gao N., Li Y., Li J. (2020). Long non-coding RNAs: the regulatory mechanisms, research strategies, and future directions in Cancers. *Oncology*.

[14] Lu B., Chen X. B., Ying M. D., He Q. J., Cao J., Yang B. (2018). The role of ferroptosis in cancer development and treatment response. *Frontiers in Pharmacology*.

[15] Pan J., Zhang X., Fang X., Xin Z. (2021). Construction on of a ferroptosis-related lncRNA-based model to improve the prognostic evaluation of gastric cancer patients based on bioinformatics. *Frontiers in Genetics*.

[16] Leng X., Liu G., Wang S. (2020). LINC01272 promotes migration and invasion of gastric cancer cells via EMT. *Oncotargets and Therapy*.

[17] Ma X., Liu Y., Tian H., Zhang B., Wang M., Gao X. (2021). LINC01272 suppressed cell multiplication and induced apoptosis via regulating MiR-7-5p/CRLS1 Axis in lung cancer. *Journal of Microbiology and Biotechnology*.

[18] Sun Z., Jing C., Xiao C., Li T. (2020). Long non-coding RNA profile study identifies an immune-related lncRNA prognostic signature for kidney renal clear cell carcinoma. *Oncology*.

[19] Lopez-Campistrous A., Adewuyi E. E., Williams D. C., McMullen T. P. W. (2021). Gene expression profile of epithelial-mesenchymal transition mediators in papillary thyroid cancer. *Endocrine*.

[20] Cao J. Y., Dixon S. J. (2016). Mechanisms of ferroptosis. *Cellular and Molecular Life Sciences*.

[21] Su Y., Zhao B., Zhou L. (2020). Ferroptosis, a novel pharmacological mechanism of anti-cancer drugs. *Cancer Letters*.

[22] Yao N., Zuo L., Yan X. (2022). Systematic analysis of ferroptosis-related long non-coding RNA predicting prognosis in patients with lung squamous cell carcinoma. Translational Lung. *Cancer Research*.

[23] He J., Zhou M., Yin J. (2021). METTL3 restrains papillary thyroid cancer neutrophil infiltration. *Molecular Therapy*.

