# Supplementary figures and images for: Identification of Ferroptosis-Associated Long Noncoding RNA Prognostic Model and Tumor Immune Microenvironment in Thyroid Cancer
Source: J Immunol Res. 2022 Jul 20;2022:5893998. doi: 10.1155/2022/5893998 (PMC9338734; doi:10.1155/2022/5893998)

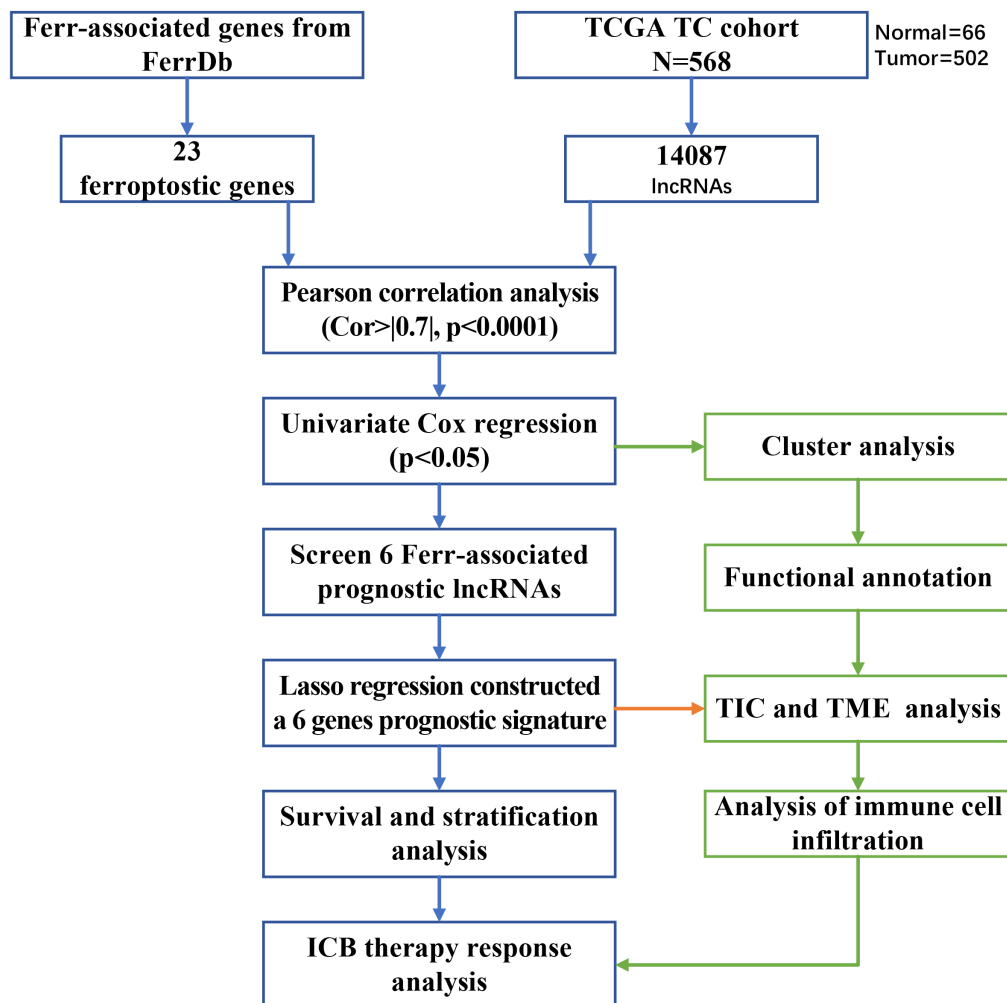

Supplement: Supplementary 1 — Additional file 1: Figure S1: the flowchart of our study. [file 5893998.f1.pdf]

a

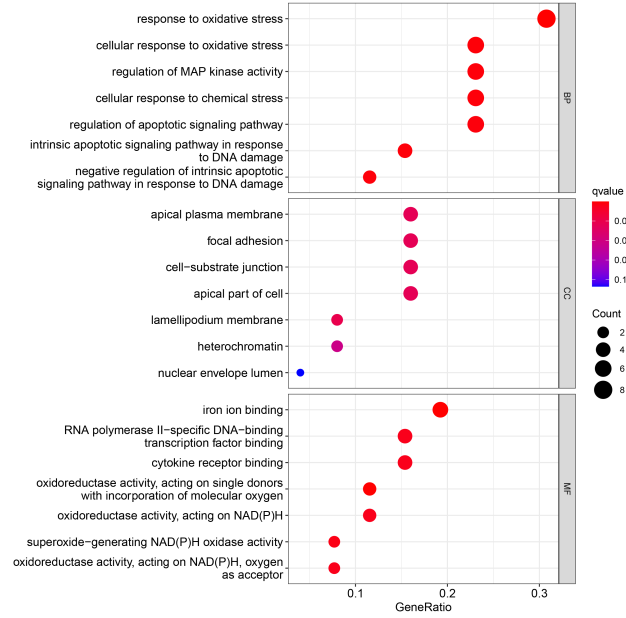

b

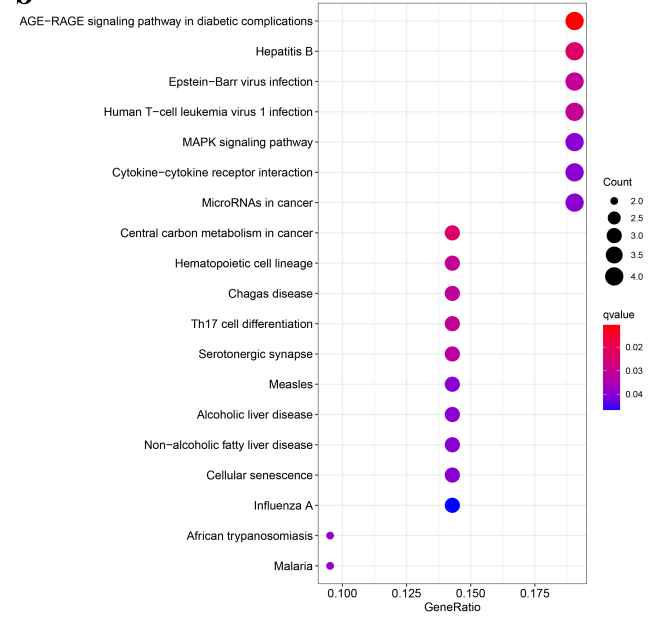

c

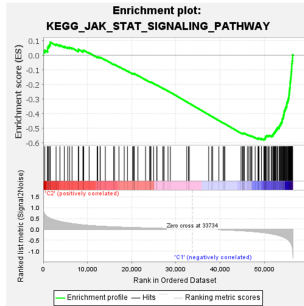

d

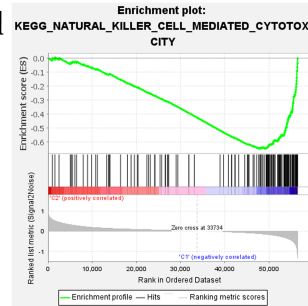

e

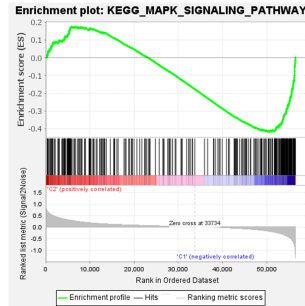

f

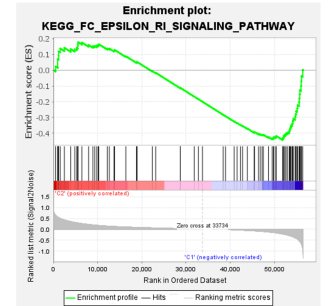

g

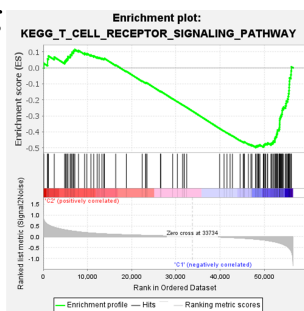

h

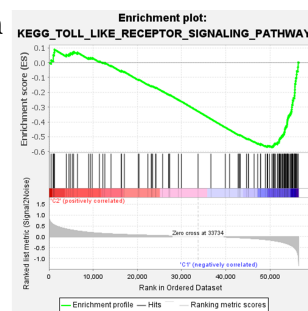

i

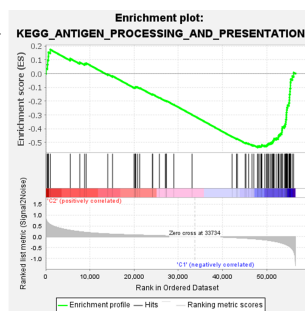

j

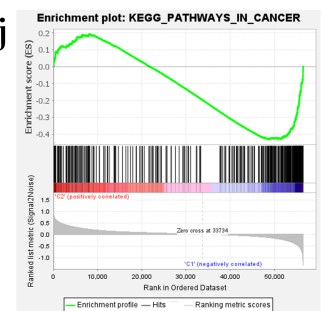

Supplement: Supplementary 2 — Additional file 2: Figure S2: functional enrichment analysis. (a, b) GO and KEGG analyses for ferroptosis-associated differentially expressed genes. (a) GO and (b) KEGG. (c–j) Several abundant immunologic characteristics of cluster1 and cluster2. [file 5893998.f2.pdf]

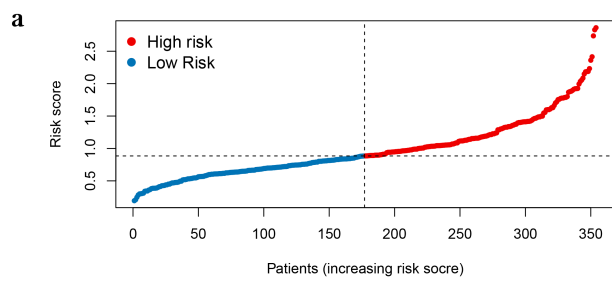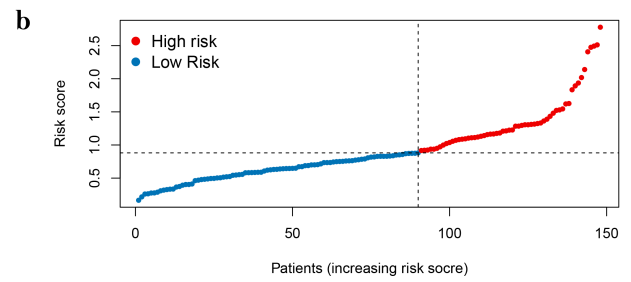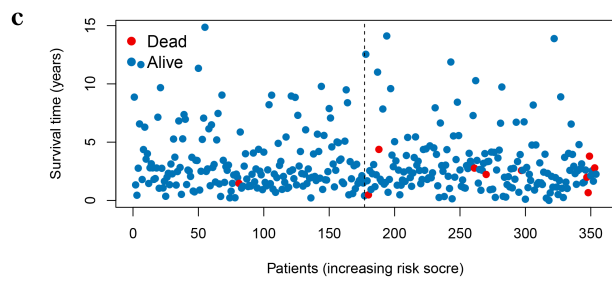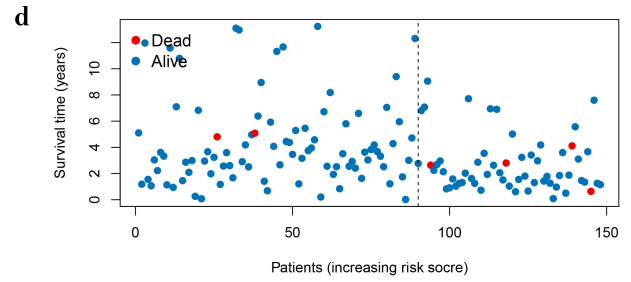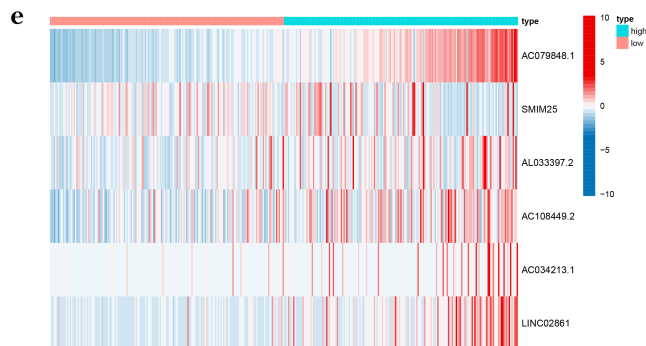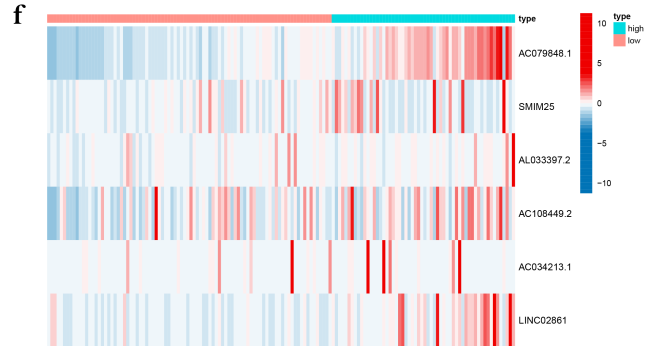

Supplement: Supplementary 6 — Additional file 6: Figure S3: identifications of prognostic gene signatures for the training set and test set, respectively. (a, b) Risk score distribution of TC patients in the training and test groups. (c, d) The scatter plot about the correlation between survival time and risk score. (e, f) The heatmap of six Ferr-associated lncRNAs in high- and low-risk TC patients. [file 5893998.f6.pdf]

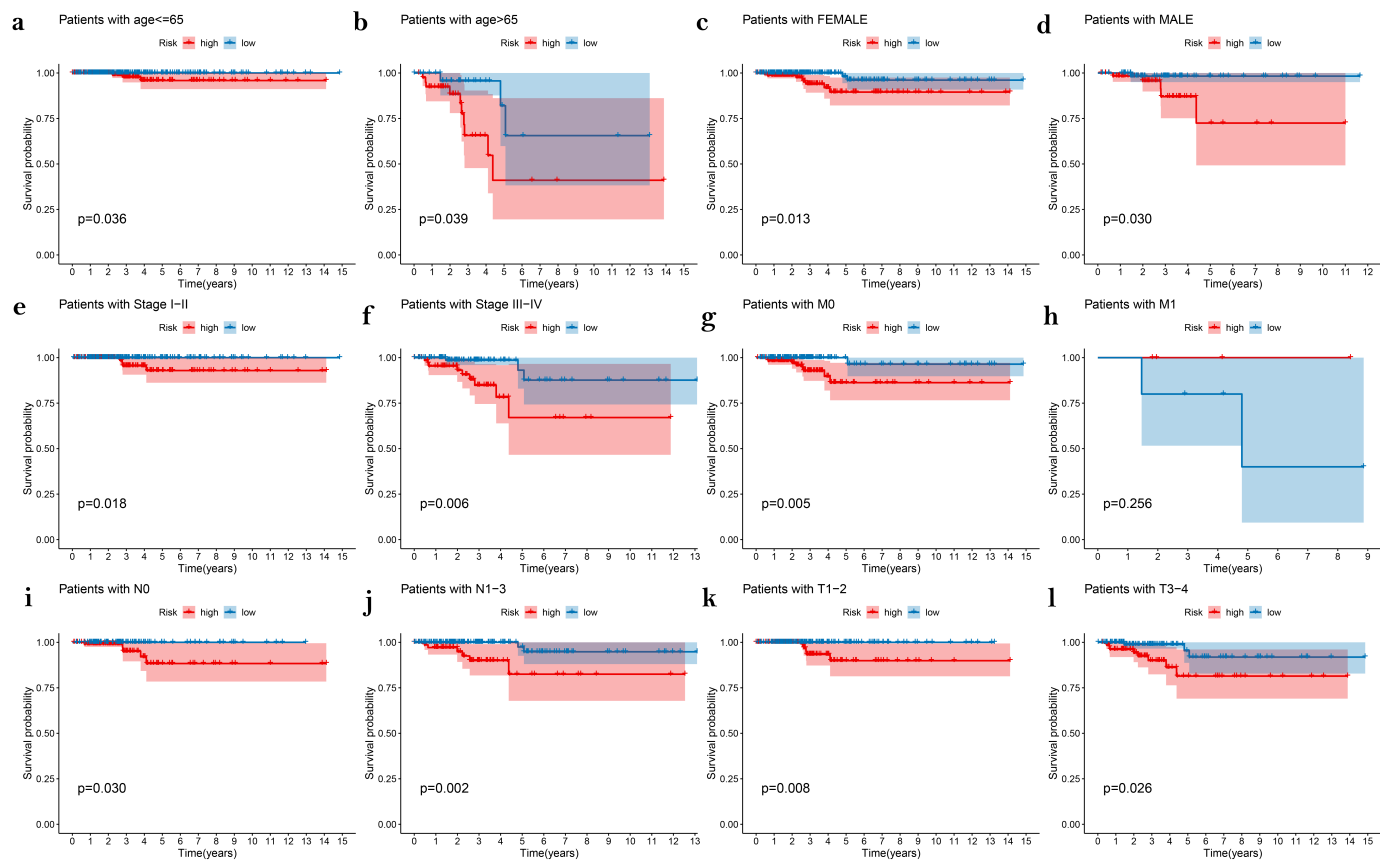

Supplement: Supplementary 7 — Additional file 7: Figure S4: the survival curves of stratification analysis. (a, b) Age. (a) ≤65 and (b) >65. (c, d) Gender. (c) Female and (d) male. (e–i) Tumor stage. (e) Stages I-II and (f) stages III-IV, (g) M0 and (h) M1, (i) N0 and (j) N1-3, (k) T1-2, and (l) T3-4. [file 5893998.f7.pdf]

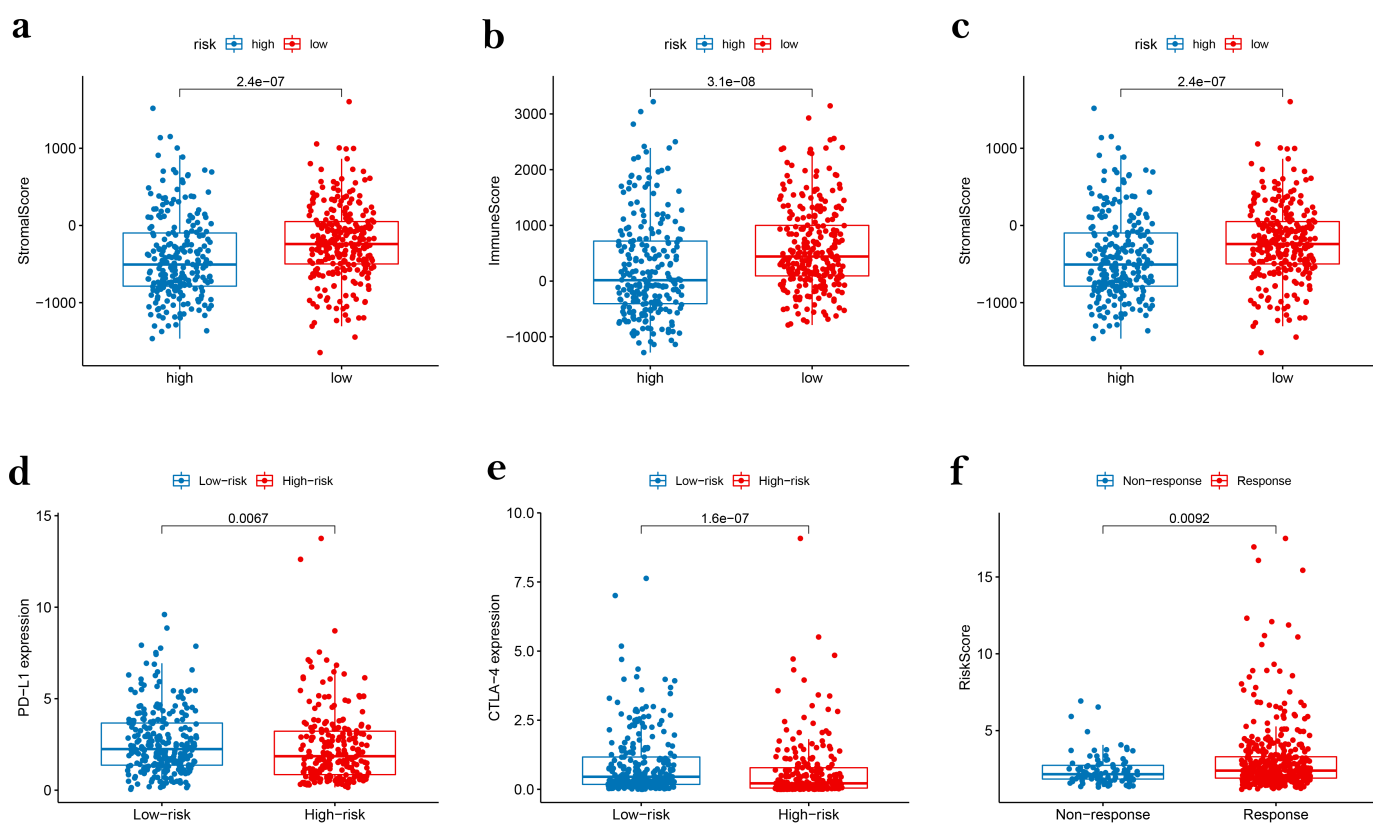

Supplement: Supplementary 8 — Additional file 8: Figure S5: tumor purity and ICB therapy response analysis. (a–c) Scattered box diagrams of immune scores, stromal scores, and ESTIMATE scores in two groups. (d, e) The differential expression analysis of two key immune checkpoints (PD-1 and CTLA4) in low- and high-risk groups. (f) The relationship between risk score and immune checkpoint blockade (ICB) therapy response. [file 5893998.f8.pdf]

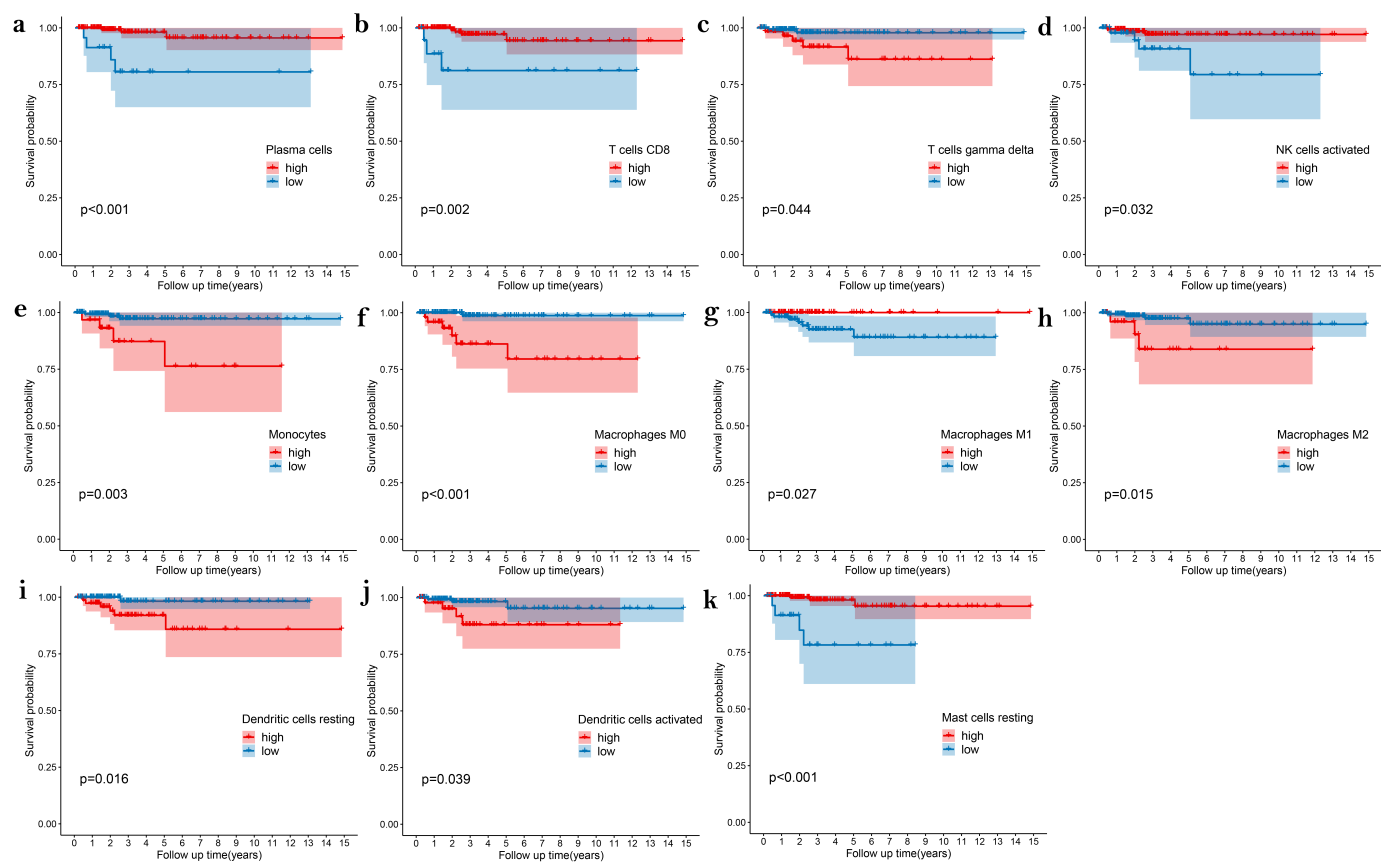

Supplement: Supplementary 9 — Additional file 9: Figure S6: the survival curves of TIICs for high- and low-risk TC patients. (a) Plasma cells, (b) T cell CD8, (c) T cell gamma delta, (d) NK cells activated, (e) monocytes, (f) macrophage M0, (g) macrophage M1, (h) macrophage M2, (i) dendritic cells resting, (j) dendritic cells activated, and (k) mast cells resting. [file 5893998.f9.pdf]
