# Supplementary material for: Identification of Ferroptosis-Associated Long Noncoding RNA Prognostic Model and Tumor Immune Microenvironment in Thyroid Cancer
Source: J Immunol Res. 2022 Jul 20;2022:5893998. doi: 10.1155/2022/5893998 (PMC9338734; doi:10.1155/2022/5893998)
Supplement: Supplementary 3 — Additional file 3: Table S1: the eleven ferroptosis-associated prognostic lncRNAs. [file 5893998.f3.docx]

| **Table S1. The eleven ferroptosis-associated prognostic lncRNAs.** | | | | |
| --- | --- | --- | --- | --- |
| **Ferr-associated lncRNA** | **HR** | **HR.95L** | **HR.95H** | **P value** |
| BX322562.1 | 1.4265 | 1.0900 | 1.8668 | 0.0096 |
| AC079848.1 | 2.7378 | 1.2824 | 5.8450 | 0.0092 |
| SMIM25 | 1.4070 | 1.1885 | 1.6656 | 0.0001 |
| AL133367.1 | 1.7308 | 1.0829 | 2.7662 | 0.0219 |
| AL033397.2 | 2.1711 | 1.0995 | 4.2869 | 0.0255 |
| AC108449.2 | 1.6404 | 1.0262 | 2.6222 | 0.0386 |
| RNF213-AS1 | 3.4898 | 1.4403 | 8.4555 | 0.0056 |
| AC034213.1 | 1.3192 | 1.0335 | 1.6837 | 0.0261 |
| LINC02345 | 3.9586 | 1.8192 | 8.6140 | 0.0005 |
| DPP4-DT | 0.1712 | 0.0492 | 0.5959 | 0.0055 |
| LINC02861 | 1.2834 | 1.0452 | 1.5757 | 0.0172 |
