# Supplementary material for: Identification of Ferroptosis-Associated Long Noncoding RNA Prognostic Model and Tumor Immune Microenvironment in Thyroid Cancer
Source: J Immunol Res. 2022 Jul 20;2022:5893998. doi: 10.1155/2022/5893998 (PMC9338734; doi:10.1155/2022/5893998)
Supplement: Supplementary 4 — Additional file 4: Table S2: the coefficients of the LASSO regression model in this study. [file 5893998.f4.pdf]

Table S2. The coefficients of LASSO regression model in this study.

Long noncoding RNA	Coefficients
AC079848.1	0.763010059541075
SMIM25	0.129275968931435
AL033397.2	0.330822322642666
AC108449.2	0.000622167544167072
AC034213.1	0.107171556233424
LINC02861	0.0766489800054426
