# Supplementary material for: Identification of Ferroptosis-Associated Long Noncoding RNA Prognostic Model and Tumor Immune Microenvironment in Thyroid Cancer
Source: J Immunol Res. 2022 Jul 20;2022:5893998. doi: 10.1155/2022/5893998 (PMC9338734; doi:10.1155/2022/5893998)
Supplement: Supplementary 5 — Additional file 5: Table S3: the baseline of training and testing sets. [file 5893998.f5.pdf]

Table S3. Baseline of training and testing sets.

	overall	train	test	p.overall
	N=502	*N=354*	*N=148*	
futime (mean (SD))	3.3 (2.7)	3.2 (2.6)	3.7 (3.0)	0.061
fustat (%):				0.577
0	486 (96.8)	344 (97.2)	142 (95.9)	
1	16 (3.2)	10 (2.8)	6 (4.1)	
Age (mean (SD))	47.3 (15.8)	46.9 (15.7)	48.5 (16.2)	0.299
Gender (%):				0.826
Female	367 (73.1)	260 (73.4)	107 (72.3)	
Male	135 (26.9)	94 (26.6)	41 (27.7)	
Stage (%):				0.622
Stage I	281 (56.0)	194 (54.8)	87 (58.8)	
Stage II	52 (10.4)	35 (9.9)	17 (11.5)	
Stage III	112 (22.3)	84 (23.7)	28 (18.9)	
Stage IV	55 (11.0)	40 (11.3)	15 (10.1)	
unknow	2 (0.4)	1 (0.3)	1 (0.7)	
T (%):				0.858
T1	143 (28.5)	98 (27.7)	45 (30.4)	
T2	164 (32.7)	119 (33.6)	45 (30.4)	
T3	170 (33.9)	120 (33.9)	50 (33.8)	
T4	23 (4.6)	16 (4.5)	7 (4.7)	
unknow	2 (0.4)	1 (0.3)	1 (0.7)	
M (%):				0.771
M0	282 (56.2)	194 (54.8)	88 (59.5)	
M1	9 (1.8)	7 (2.0)	2 (1.4)	
MX	210 (41.8)	152 (42.9)	58 (39.2)	
unknow	1 (0.2)	1 (0.3)	0 (0.0)	
N (%):				0.969
N0	229 (45.6)	162 (45.8)	67 (45.3)	
N1	223 (44.4)	156 (44.1)	67 (45.3)	
NX	50 (10.0)	36 (10.2)	14 (9.5)	
